# Cadherin-5: a biomarker for metastatic breast cancer with optimum efficacy in oestrogen receptor-positive breast cancers with vascular invasion

**DOI:** 10.1038/bjc.2016.66

**Published:** 2016-03-24

**Authors:** Simon A Fry, Claire E Robertson, Ruth Swann, Miriam V Dwek

**Affiliations:** 1 Faculty of Science and Technology, Department of Molecular and Applied Biosciences, University of Westminster, 115 New Cavendish Street, London W1W 6UW, UK; 2 Faculty of Science and Technology, Department of Human and Health Sciences, University of Westminster, 115 New Cavendish Street, London W1W 6UW, UK

**Keywords:** breast cancer, metastasis, biomarker, glycosylation, cadherin-5, ER positive, vascular invasion

## Abstract

**Background:**

A glycoproteomic study has previously shown cadherin-5 (CDH5) to be a serological marker of metastatic breast cancer when both protein levels and glycosylation status were assessed. In this study we aimed to further validate the utility of CDH5 as a biomarker for breast cancer progression.

**Methods:**

A nested case–control study of serum samples from breast cancer patients, of which *n*=52 had developed a distant metastatic recurrence within 5 years post-diagnosis and *n*=60 had remained recurrence-free. ELISAs were used to quantify patient serum CDH5 levels and assess glycosylation by *Helix pomatia* agglutinin (HPA) binding. Clinicopathological, treatment and lifestyle factors associated with metastasis and elevated biomarker levels were identified.

**Results:**

Elevated CDH5 levels (*P*=0.028) and ratios of CDH5:HPA binding (*P*=0.007) distinguished patients with metastatic disease from those that remained metastasis-free. Multivariate analysis showed that the association between CDH5:HPA ratio and the formation of distant metastases was driven by patients with oestrogen receptor (ER+) positive cancer with vascular invasion (VI+).

**Conclusions:**

CDH5 levels and the CDH5 glycosylation represent biomarker tests that distinguish patients with metastatic breast cancer from those that remain metastasis-free. The test reached optimal sensitivity and specificity in ER-positive cancers with vascular invasion.

Serum tumour markers are economic, non-invasive tests that can be used to aid diagnosis, to monitor disease progression and patient response to treatment. In order for oncologists to tailor individual treatment strategies, breast cancer patients with the highest risk of developing metastatic disease need to be classified to identify those most likely to benefit from adjuvant therapy, whilst minimising the number of women receiving unnecessary therapies. Two of the most widely utilised breast cancer biomarkers are cancer antigen 15.3 (CA15.3) and carcinoembryonic antigen (CEA), measured after primary breast cancer treatment for the detection of recurrent disease or metastasis (Sturgeon *et al*, 2008, 2009). However, the most appropriate use of tumour markers for monitoring breast cancer progression remains the subject of considerable debate in the scientific community (Mirabelli and Incoronato, 2013). Recently, new imaging techniques have been allied with serum tumour marker measurements to identify cancerous lesions non-detectable by conventional imaging modalities, for example, positron emission tomography imaging has been shown to detect breast cancer recurrence and metastasis in patients with rising levels of tumour markers such as CA15.3 (Champion *et al*, 2011; Katayama *et al*, 2012). This provides a renewed impetus in the search for serum markers for breast cancer which may allay the criticism current markers have attracted, whilst increasing the diagnostic ability of imaging techniques.

Many serological cancer biomarkers are glycoproteins. Glycosylation is one of the most frequent post translational modifications to occur on proteins (Khoury *et al*, 2011), but in the majority of diagnostic tests protein levels are monitored whilst the glycan moiety, and therefore biological information possibly pertaining to pathology, is ignored. As aberrant glycosylation is known to accompany malignant transformation, this offers potential for refinement of cancer serum biomarker tests (Kuzmanov *et al*, 2013). In an attempt to address the under-exploitation of glycosylation for breast cancer biomarker discovery, we previously employed a glycoproteomic approach and reported many serum proteins with altered glycosylation in metastatic breast cancer. Using this approach, cadherin-5 (CDH5) emerged as a novel biomarker for metastatic breast cancer (Fry *et al*, 2013), when assessed by ELISAs that incorporate measurements of glycosylation status, evaluated by binding of the lectin *Helix pomatia* agglutinin (HPA) known to detect poor prognosis metastatic cancer (Schumacher and Adam, 1997; Mitchell and Schumacher, 1999; Dwek *et al*, 2001).

In this study we aimed to further determine the utility of CDH5 as a biomarker for breast cancer progression by identifying clinicopathological, treatment and lifestyle factors associated with metastasis and elevated biomarker levels. Serum samples from breast cancer patients were analysed to obtain measurements of CDH5 and to determine the glycosylation status as monitored by HPA binding. The CDH5:HPA test emerged as a novel means for detection of metastatic breast cancer in patients with ER-positive tumours that have infiltrated the vasculature.

## Materials and methods

### Patient samples

Serum samples were routinely collected as part of the DietCompLyf study (Swann *et al*, 2013) approved by the UCL/UCLH Committees on the Ethics of Human Research (ref. 96/3433 and 98/0090). All patients consented to participation in the study. *n*=2808 breast cancer patients were recruited onto the study (new protocol) 9–15 months post breast cancer diagnosis, whereupon the first of five annual venipunctures was performed and blood was collected into 6 ml vacutainers (BD Bioscience, Plymouth, UK), left to stand for 1 h to clot, centrifuged at 1200 g for 15 min and the resulting supernatant aliquoted into 0.5 ml fractions and frozen at −20 °C or −70 °C. Patients (*n*=207) developed distant metastases by October 2012, of which *n*=120 had available serum samples from year 1 and at least one other year of the DietCompLyf study. Serum samples were analysed from 2 patient groups; those with no sign of distant metastatic recurrence (NSR) 48 months (±3 months) post recruitment (60 months ±3 months post-diagnosis), and those with distant metastatic recurrence (REC) within the same time period. NSR and REC patient groups were selected according to their age at diagnosis, tumour size, grade, lymph node, oestrogen receptor, progesterone receptor and human epidermal growth factor receptor 2 statuses, Table 1.

### Sample size calculation

The CDH5 levels recorded previously (Fry *et al*, 2013) for patients with either NSR (m1) or REC (m2) were used to estimate the sample size required in each group using the following equation: *n*=2 × (sd)^2^ × (z*α*+z*β*)^2^/(m1−m2)^2^ using multipliers based on significance level (z_*α*_) and power (z_*β*_; Suresh and Chandrashekara, 2012). This identified a sample size of 92 samples per group to achieve 95% significance and 90% power. After analysis of *n*=60 NSR and *n*=52 REC samples, statistical significance was reached for the CDH5 ELISAs and to preserve the DietCompLyf sample bank no further serum testing was performed.

### CDH5 ELISA

Ninety-six-well plates (Immuno Maxisorp, Thermo Scientific, Loughborough, UK) were coated with 50 *μ*l mouse monoclonal anti-human CDH5 (MM0012-8A03, Insight Biotechnology, Middlesex, UK) diluted to 0.2 *μ*g ml^−1^ in carbonate/bicarbonate buffer, pH 9.6, by overnight incubation at 4 °C. All subsequent steps were undertaken at room temperature on an orbital shaker. Wells were washed five times with 200 *μ*l phosphate-buffered saline/0.1% (v/v) Tween 20 (PBS/T), blocked by addition of 200 *μ*l Carbofree Solution (Vector Labs, Peterborough, UK) for 30 min and then washed a further five times with 200 *μ*l PBS/T. Patient sera were diluted 1 in 50 and 1 in 250 in PBS. 25 *μ*l of each dilution was added to wells in duplicate followed by addition of 50 *μ*l Carbofree solution to each well. Plates were incubated for 2 h, washed five times with 200 *μ*l PBS/T, and then 50 *μ*l of biotinylated goat polyclonal anti-human CDH5 (N-14, Insight Biotechnology) prepared at 0.5 *μ*g ml^−1^ in Carbofree solution was added for 1 h. Wells were washed four times with 200 *μ*l PBS/T and 50 *μ*l of streptavidin conjugated to poly-horse radish peroxidase (Thermo Scientific) diluted 1 : 4000 in Carbofree Solution was added for 1 h. Wells were washed three times with 200 *μ*l PBS/T followed by a further three times with deionised water before addition of 100 *μ*l 3,3′,5,5′-tetramethylbenzidine (TMB) microwell peroxidase substrate (Insight Biotechnology). The reaction was monitored using a Dynatech MRX plate reader (Dynatech Medical Products, Alexandria, VA, USA) and quenched by addition of 100 *μ*l 1 M phosphoric acid when the absorbance measured at 630 nm reached ∼0.6 absorbance units. Following quenching the absorbance was read at 450 nm in a Wallac 1420 Victor 2 plate reader (Perkin Elmer, Beaconsfield, UK).

### Refined CDH5 ELISA to measure CDH5 glycosylation status

Ninety-six-well plates were coated, incubated and then washed as described in the previous section. Seventy-five microlitre of cold 20 mM periodic acid was added to each well for 30 min at 4 °C. Following five washes with 200 *μ*l PBS/T plates were blocked and washed as before. Patient serum was diluted 1 in 25 and 1 in 125 in PBS. Twenty-five microlitre of each dilution was added to wells in duplicate, before addition of 50 *μ*l Carbofree solution to each well. Plates were incubated for 2 h, washed five times with 200 *μ*l PBS/T, and then 50 *μ*l of biotinylated HPA (Sigma Aldrich, Dorset, UK) at 10 *μ*g ml^−1^ in Carbofree solution was added for 1 h. Wells were washed as before and 50 *μ*l of streptavidin conjugated to poly-horse radish peroxidase diluted 1 : 1000 in Carbofree solution was added for 1 h. Wells were washed three times with 200 *μ*l PBS/T followed by a further three times with deionised water before addition of 100 *μ*l TMB microwell peroxidase substrate. The reaction was monitored and the absorbance read as described above.

### Quantification of CA15.3 and CEA

CA15.3 and CEA serum measurements were made by The Doctors Laboratory (TDL, London, UK), an independent clinical pathology accredited laboratory. CA15.3 and CEA were quantified by the Roche Modular method.

### VEGF ELISA

Serum VEGF levels were measured using a human ‘VEGF DuoSet ELISA development system kit' (R&D Systems, Oxon, UK) according to the manufacturer's instructions with the following alterations: 100 *μ*l streptavidin-HRP from the kit was replaced with streptavidin conjugated to poly-horseradish peroxidase (Thermo Scientific) diluted 1 : 4000 in reagent diluent; patient sera were diluted 1 : 10 in reagent diluent and assayed in duplicate; the uppermost point on the standard curve was 500 pg ml^−1^ (seven point standard curve). These alterations were made to enhance the sensitivity of the assay to minimise the volume of patient serum required.

### Sample analysis

For the CDH5 ELISAs described above, two-fold serial dilutions of pooled reference serum with known CDH5 concentration were used to produce standard curves. Standard curves were measured in duplicate on each 96-well plate, with the limit of detection defined as the mean average blank reading (wells containing all reagents but no serum) plus two s.d.'s. Levels of CDH5 and HPA binding for the patient samples were determined by interpolation against the standard curves (after subtraction of blank readings from the standard curves and from the patient serum absorbance values). Three quality control samples were assayed on each plate to enable inter-assay comparison. The highest absorbance reading falling within the linear detection range on the standard curves was used to infer the relative protein levels, taking into account any dilution factor.

The CDH5, HPA, CDH5:HPA ratio, CA15.3, CEA and VEGF values were analysed as continuous variables using the Mann–Whitney test to assess biomarker discrimination between NSR and REC samples. Univariate analysis using the Kruskal–Wallis or Mann–Whitney test was employed to assess associations between patient CDH5:HPA ratios and clinicopathological, treatment and lifestyle factors. The same tests were used to establish whether an association between CDH5:HPA ratios and any of these factors was evident when comparing NSR patients with REC patients.

Using multiple logistic regression models, the clinicopathological variables shown in table one were built into models in a stepwise manner to determine their capability in prediction of distant recurrence. The sensitivity, specificity and the negative and positive predictive values (NPV/PPV) were determined.

## Results

### CDH5–HPA ELISAs

**Figure 1:**
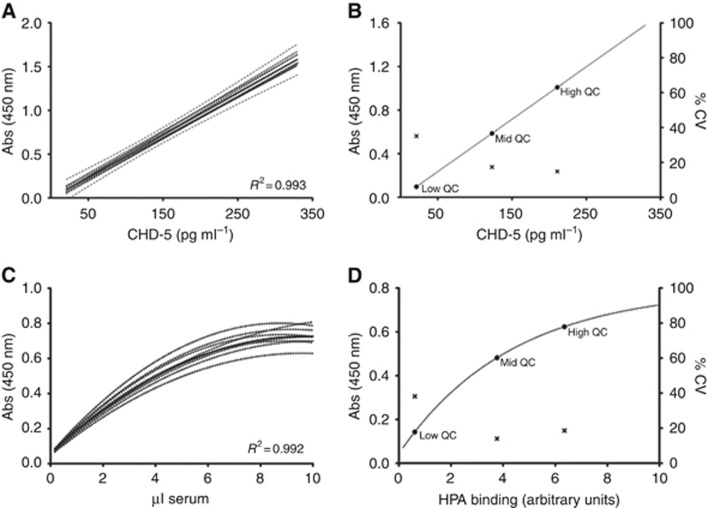
**CDH5 ELISA standard curves and quality control samples.** (**A**) CDH5 standard curves (broken lines) with mean values indicated. (**B**) CDH5 QC samples, with inter-assay variability on right hand axis. (**C**) Refined ELISA (for HPA binding measurement) standard curves (broken lines) with mean value indicated. (**D**) Refined ELISA QC samples, with inter-assay variability on right hand axis.

Levels of CDH5 were determined in breast cancer serum samples by interpolation from standard curves showing intra- and inter-assay coefficients of variation of 6.8% and 15.3%, respectively. The highly reproducible nature of the CDH5 assay is further evidenced by them all falling within the 95% confidence interval of the combined mean of all curves, *R*^2^=0.993 (Figure 1A). To allow inter-assay comparisons, three quality control serum samples were included on each 96-well plate, specifically selected to represent a range of CDH5 concentrations (Figure 1B). The linear working range of the assay was 21–330 pg ml^−1^. As expected, the quality control sample with the lowest CDH5 concentration showed the greatest degree of variability, accordingly, the patient sample dilution of the greatest optical density falling within the linear range of the standard curve was used to determine the CDH5 levels (Figure 1B). In all, 52 out of the 112 patient samples analysed had detectable CDH5 levels in both serum dilutions assayed. When the data were normalised to equivalent concentrations of serum, the coefficient of variation between sample dilutions was 13.5% and general agreement between CDH5 concentrations measured at each dilution (1 in 50 and 1 in 250) was good (Spearman rank correlation coefficient=0.85, *P*<0.0001; Supplementary Figure 1A).

The ELISA was refined by pre-treating the capture antibody with periodic acid to oxidise vicinal diols of the glycans to aldehyde groups, rendering the capture antibody non-reactive with HPA. The refined ELISA therefore was used to measure the glycosylation status of the CDH5 protein. The pooled reference serum was used to generate standard curves: mean *R*^2^=0.992 (Figure 1C). Intra- and inter-assay coefficients of variation were 5.9% and 12.2%, respectively. Again, three quality control samples were incorporated on each plate to assess assay reproducibility. One NSR and one REC patient had non-detectable HPA binding levels at both of the serum dilutions assayed, and 66 out of 110 patient samples analysed had HPA levels that were detectable at both serum dilutions. Agreement between sample dilutions was not quite as good as observed in the CDH5 ELISA, with an average coefficient of variation of 24.9% after normalising the data to equivalent volumes of serum, Spearman correlation=0.78, *P*<0.0001 (Supplementary Figure 1B).

### Biomarker levels discriminate between NSR and REC

**Figure 2:**
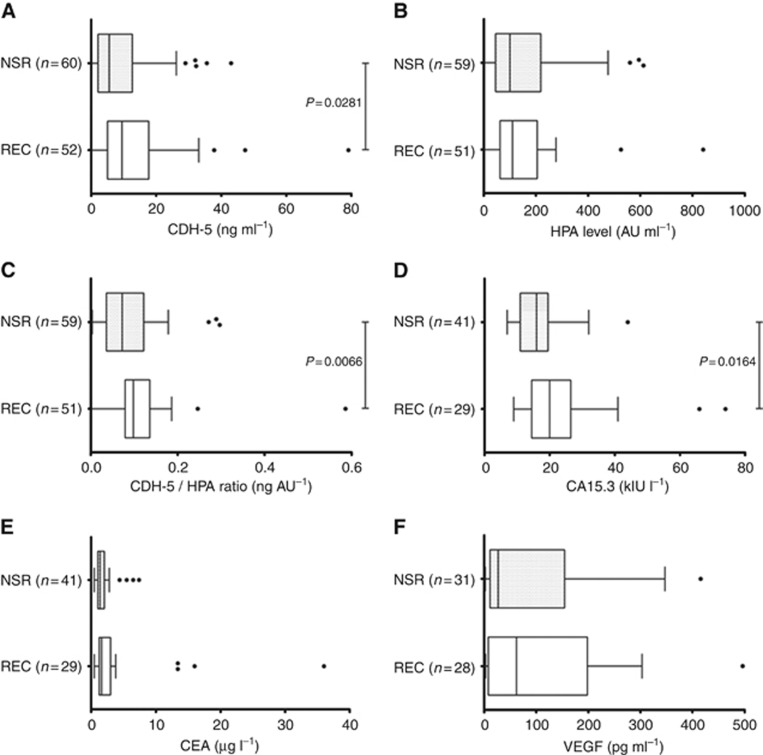
**Box plots showing biomarker levels in patients with either NSR or REC.** (**A**) CDH; (**B**) HPA binding to (antibody) captured CDH; (**C**) Ratio of CDH5:HPA; (**D**) CA15.3; (**E**) CEA; (**F**) VEGF. The whiskers represent the data within 1.5 IQR of the upper and lower quartiles. Outliers are indicated by dots. Statistical significance indicated.

Table 1 presents data showing that patients with recurrent breast cancer had significantly higher levels of serum CDH5 compared with patients that remained recurrence-free for 5 years (*P*=0.028, Figure 2A) and whilst HPA binding was comparable between the 2 sample groups (Figure 2B) the CDH5:HPA ratio was also significantly elevated (*P*=0.007) in patients with REC compared with patients with NSR (Figure 2C).

CA15.3 and CEA are the most widely investigated serum biomarkers for breast cancer (Duffy, 2006), both of which show elevated levels in a proportion of patients with metastatic disease (Cheung *et al*, 2000). Of the initial cohort of 112 breast cancer patient serum samples analysed, 70 (*n*=41 NSR, *n*=29 REC) had sufficient volume remaining for measurements of CA15.3 and CEA. Figure 2D shows that patients with recurrent breast cancer had significantly higher levels of serum CA15.3 compared with patients that remained recurrence-free for 5 years (*P*=0.0164). CEA measurements did not distinguish NSR from REC samples (Figure 2E).

VEGF has been shown to be associated with tumour angiogenesis (Rykala *et al*, 2011). To assess whether the CDH5 levels detected in the patient serum samples were associated with an increased level of circulating VEGF, and potentially, therefore, tumour angiogenesis, a commercially available ELISA kit was adapted to enable measurement of 3.9–500 pg ml^−1^ VEGF in serum. Approximately half of the serum samples from the breast cancer patients had detectable levels of serum VEGF, however, no significant difference (*P*=0.75) in the VEGF levels were observed between the two patients groups: NSR and REC (Figure 2F). Similarly, there was no statistically significant association between the levels of serum VEGF and either the CDH5, HPA or CDH5:HPA ratio values, (Spearman's rank correlation, *P*>0.05).

### Statistical analysis of clinicopathological, treatment and lifestyle factors

**Figure 3:**
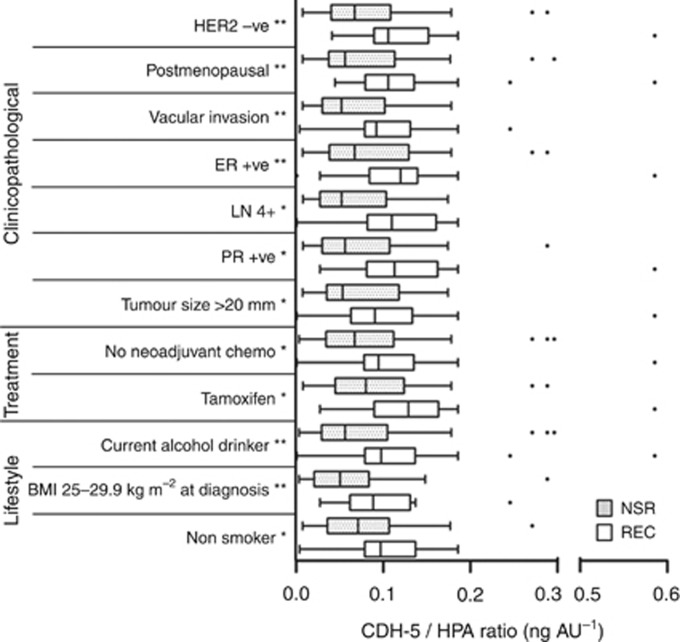
**Box plots showing clinicopathological, treatment and lifestyle factors (listed from most to least significant) that distinguish NSR and REC patients when their ratio of CDH5:HPA binding is assessed.** The whiskers represent data within 1.5 IQR of the upper and lower quartiles. Outliers are indicated by dots. Significance identified using Mann–Whitney or the Kruskal–Wallis tests are indicated **P<0.01, *P<0.05.

When all 112 patients were analysed, no statistically significant differences in the distribution of CDH5:HPA ratios were found for any clinicopathological, treatment or lifestyle factors (Table 1). The CDH5:HPA ratios were significantly increased (Mann–Whitney or the Kruskal–Wallis tests) in patients who had developed recurrences (*n*=51) compared with those with no sign of recurrence (*n*=59) when tumour size, vascular invasion, lymph node involvement, menopausal and ER, PR and HER2 status, treatment (Tamoxifen, neoadjuvant chemotherapy) and lifestyle factors (alcohol consumption, BMI and smoking status) were considered, Figure 3.

The influence of the following clinicopathological parameters on the chance of developing distant metastasis was investigated: tumour size (continuous variable), tumour grade, vascular invasion, lymph node status, ER, PR, HER2 status and CDH5:HPA ratio. The CDH5:HPA ratio data showed significant positive skew, and thus was considered as a categorical variable (according to tertiles: low=0.007–0.062 ng AU^−1^ (*n*=36), mid=0.062–0.117 ng AU^−1^ (*n*=37), high=0.117–0.586 ng AU^−1^ (*n*=37)) in the logistic regression model which predicted breast cancer metastasis in a statistically significant manner (*P*=0.036). The accuracy of this model for classification of breast cancer metastasis was 72.6% specificity and sensitivity was 74.3% and 70.4%, respectively (Supplementary Table 1). On examination, ER status (*P*=0.024), CDH5:HPA ratio (*P*=0.030) and vascular invasion (*P*=0.038) were all statistically significant suggesting that the CDH5:HPA ratio is of greatest utility as a predictor of breast cancer metastasis in ER positive tumours that have vascular invasion.

### Sensitivity and specificity

**Figure 4:**
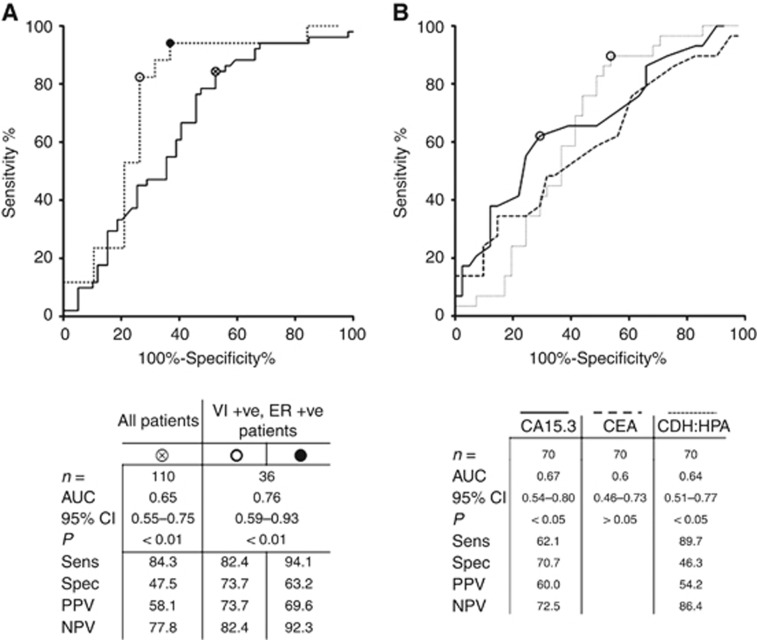
**ROC curves plotted to calculate sensitivity, specificity, PPV and NPV.** (**A**) CDH5:HPA ratio for all patients (solid line) and for vascular invasion positive, ER-positive patients (broken line). (**B**) Comparison of CA15.3, CEA and CDH:HPA.

A receiver operating characteristic (ROC) curve was plotted and the optimal sensitivity, specificity, PPV and NPV of CDH5:HPA ratio for the detection of metastatic breast cancer was calculated with data from 110 samples (Figure 4A). Using the Youden index to apply equal emphasis to minimise false positive and negative readings, the CDH5:HPA ratio distinguished NSR from REC serum samples with 84.3% specificity and 47.5% sensitivity, with corresponding PPV and NPV values of 58.1% and 77.8%, respectively. When the results from patients with ER-positive tumours with vascular invasion were considered, the CDH5:HPA ratio was able to further discriminate between NSR and REC breast cancer patients. In this analysis the sensitivity and specificity reached 82.4% and 73.7%, respectively, the PPV was 73.7% and the NPV was 82.4% (Figure 4A). The sensitivity of the CDH5:HPA ratio assay was maximal at 94.1% (NPV=92.3%) with a concomitant reduction in specificity to 63.2% (PPV=69.6%).

ROC analysis of the sub-cohort of 70 patients from which serum CA15.3 was quantified revealed that CA15.3 is a more specific marker of metastatic breast cancer than CDH:HPA (70.7% and 46.3%, respectively), whilst CDH:HPA is more sensitive (89.7% *vs* 62.1%, Figure 4B). Combining CA15.3 and CDH:HPA ratio values did not significantly increase the area under the ROC curve (AUC) relative to AUCs obtained for CA15.3 or CDH:HPA individually (data not shown).

### CDH5:HPA ratio tertiles

**Figure 5:**
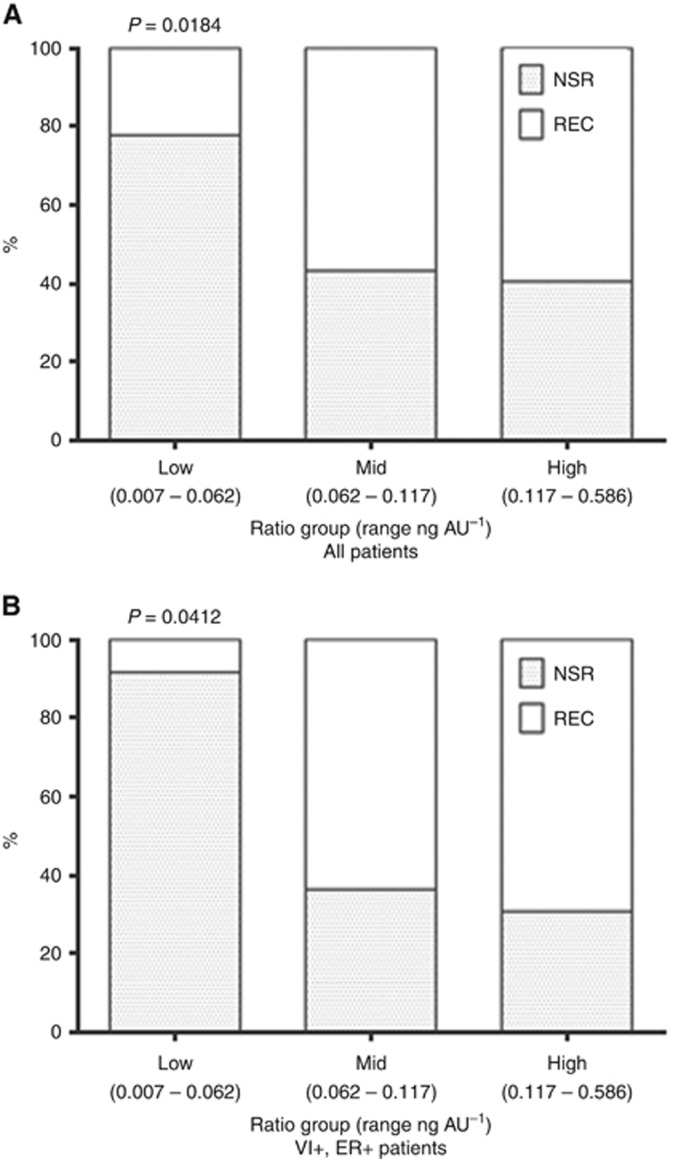
**Distribution of all patients (**A**) and vascular invasion positive, ER-positive patients (**B**) with metastatic breast cancer according to tertiles of CDH5:HPA ratio.** *χ*^2^ *P*-values indicated.

When taken as a continuous variable the CDH5:HPA ratio did not correlate with time to, or the site of, metastasis (data not shown). However, when considered as a categorical variable 78% of breast cancer patients with a CDH5:HPA ratio in the low category (*n*=36) were metastasis-free after 5 years (*χ*^2^(1, *N*=36)=5.56, *P*=0.0184). This figure fell to 43% and 40% for patients with CDH5:HPA ratios in the mid and high categories, respectively. When considering only those patients with ER-positive tumours with vascular invasion, 92% of those in the low CDH5:HPA ratio category remained metastasis-free after 5 years (*χ*^2^(1, *N*=12)=4.17, *P*=0.0412; Figure 5).

## Discussion

A previous glycoproteomic investigation identified CDH5 as a novel biomarker of metastatic breast cancer. An ELISA-based methodology was developed to enable the CDH5 protein levels to be measured in the serum of breast cancer patients alongside assessment of CDH5 glycosylation status using HPA as the glycan recognition molecule (Fry *et al*, 2013). The assays were employed in this study of a new set of serum samples from the DietCompLyf cohort (Swann *et al*, 2013). Comparable results to those obtained previously were observed, further strengthening the evidence for CDH5 as a biomarker of breast cancer metastasis and demonstrating the importance of assessing both the protein level and glycosylation status. Both the CDH5 protein level and the CDH5:HPA ratio were significantly increased in the sera of patients with metastatic breast cancer compared to patients that remained disease free for 5 years post breast cancer diagnosis. The data from ELISAs suggests that CDH5 and CDH5:HPA ratio values may offer a lead time in the diagnosis of metastatic disease, as the average time to metastasis (from diagnosis) in this cohort of patients was 32.0 months (±3 months) post primary breast cancer diagnosis.

Serial measurements of CA15.3 have been shown to detect recurrent/metastatic disease with a lead time of 2–9 months (Duffy, 2006). Consistent with this, serum CA15.3 measurements were shown to discriminate patients with metastatic breast cancer from patients that remained disease free for 5 years. As the most commonly employed marker for breast cancer, CA15.3 is widely considered as the gold standard against which other markers must be compared. Thus, it is encouraging that measurement of serum CDH5 protein levels compared favourably to those of CA15.3 in discriminating metastatic breast cancer sera. When comparing CA15.3 to the CDH:HPA ratio values, the most specific test, least likely to lead to false positive classifications, is CA15.3. Conversely, CDH:HPA ratio values produce a more sensitive test for metastatic breast cancer.

CDH5 is a cell membrane glycoprotein found at adherens junctions where it acts as an adhesion receptor between non-proliferative endothelial cells (Giannotta *et al*, 2013). The first report of aberrant levels of CDH5 in cancer was in uveal melanoma in 2001 (reported in Breier *et al*, 2014), since then CDH5 has also been implicated in aggressive breast cancer (Parker *et al*, 2004; Labelle *et al*, 2008). A murine model of mammary carcinogenesis revealed that cells that had undergone epithelial to mesenchymal transition (EMT), a phenotypic alteration associated with metastatic dissemination (Kalluri and Weinberg, 2009), had increased tumour cell proliferation and increased cancer cell adhesion to endothelial cells associated with increased cell surface CDH5. In addition, CDH5 promoted breast cancer progression via the transforming growth factor *β* (TGF-*β*) signalling pathway (Labelle *et al*, 2008) the same pathway that promotes cell proliferation and EMT in tumours with an invasive cellular phenotype (Yilmaz and Christofori, 2009). The interaction between CDH5 and TGF-*β* receptors has been demonstrated to enhance TGF-*β* signalling in endothelial cells (Rudini *et al*, 2008). CDH5 and VEGF are recognised mediators of tumour angiogenesis, a process required for invasion and metastasis of solid tumours (Weidner *et al*, 1991), and VEGF inhibitors have been approved for the treatment of advanced cancer (Longatto Filho *et al*, 2010). In this study, serum VEGF levels did not distinguish patients with metastatic disease from patients with no sign of metastasis, consistent with a recent report in which lymphovascular invasion rather than angiogenesis was associated with poorer recurrence-free and cancer-specific survival (Mohammed *et al*, 2013), suggestive that tumour growth is dependent on angiogenesis, whilst lymphovascular invasion may be essential for metastasis.

Univariate analysis revealed that patients with recurrent breast cancer and significantly elevated CDH5:HPA ratios typically had vascular invasion, ⩾4 positive lymph nodes and a tumour >20 mm in diameter (Weigelt *et al*, 2005). Post-menopausal ER- and PR-positive breast cancer patients with metastatic disease showed significantly raised CDH5:HPA ratios compared with those who remained metastasis-free. As would be expected, the majority of the breast cancer patients studied here were treated with the hormone therapy Tamoxifen, and this was associated with an increased CDH5:HPA ratio. As the majority of newly diagnosed breast cancers are ER positive (Yasui and Potter, 1999; Anderson *et al*, 2002; Piccart-Gebhart, 2011) it is therefore encouraging that the CDH5:HPA ratio was able to discriminate ER-positive breast cancer patients with different clinical outcomes. Similarly the CDH5:HPA ratio was elevated in the serum of HER2-positive breast cancer patients who developed recurrent disease.

Alcohol consumption and obesity are modifiable factors that have previously been linked to increased breast cancer recurrence rates. Alcohol drinkers and patients with a high BMI (25–29.9 kg m^−2^) at diagnosis, as well as non-smokers, showed significantly raised CDH5:HPA ratios associated with the formation of distant metastases but the significance of these findings is unclear.

Multivariate analysis showed that the CDH5:HPA ratio best predicts breast cancer metastasis in ER-positive tumours where the tumour has invaded the vasculature (Figure 4A). Stratifying patients according to ER status and vascular invasion had negligible effect on the sensitivity or NPV of the CDH5:HPA assay, but dramatically increased the specificity and PPV. High specificity and NPV are desirable attributes for biomarkers of metastatic cancer minimising false positives and are important for optimal patient management in the clinic. Of the sample set analysed here, 74% of patients with an ER-positive primary tumour with vascular invasion were ectly identified as developing distant metastases, and 82% of patients stratified in the same way were correctly identified as remaining metastasis-free for 5 years. Taking the lower tertile of CDH5:HPA ratios (0.007–0.062 ng AU^−1^) revealed that 11 of the 12 ER-positive vascular invasion positive patients remained free of distant metastases for 5 years. Clearly, the utility of the biomarker tests described here will require further investigation using analytical methods such as Kaplan–Meier and Cox's regression to determine the relationship between biomarker levels and time to breast cancer recurrence.

In conclusion, the assays described here to measure pg ml^−1^ levels of serum CDH5 and probe CDH5 glycosylation are sensitive, accurate and reproducible. Serum CDH5 levels and CDH5:HPA ratio values showed these biomarkers to be significantly elevated in patients with metastatic breast cancer, a finding which was paralleled by measurements of CA15.3, which is a more specific but less sensitive marker than CDH:HPA ratios. Univariate analysis revealed that CDH5:HPA ratios correlated with several prognostic indicators for breast cancer metastasis. Multivariate analysis showed that patients with ER-positive cancer with vascular invasion were driving the association between the CDH5:HPA ratio and the formation of distant metastases. The CDH5:HPA ratio detected patients with recurrent breast cancer with ER-positive primary tumours containing vascular invasion with 82% sensitivity and 74% specificity. In the lower tertile of CDH5:HPA ratios, 92% of patients with ER-positive primary tumours containing vascular invasion had no sign of distant metastasis, indicating the sub-group of breast cancer patients for whom measurement of this biomarker is the most beneficial.

## Figures and Tables

**Table 1:** Descriptive covariates, showing clinicopathological, treatment and lifestyle features of patients from which the samples were taken as well as the results that were obtained for biomarker measurement

		**NSR (*****n*****=60)**		**REC (*****n*****=52)**		**Total subjects (*****n*****=112)**	
		**Mean (s.d.)**	**Range**	**Mean (s.d.)**	**Range**	**Mean (s.d.)**	**Range**
Age at diagnosis	*	52.19 (9.29)	33.45–71.76	53.21 (11.51)	31.80–74.50	52.66 (10.29)	31.80–74.50
Tumour size (mm)	*	24.14 (15.53)	2–70	28.54 (20.51)	5–110	26.15 (18.02)	2–110
Height (m)		1.63 (0.06)	1.50–1.79	1.63 (0.07)	1.47–1.80	1.63 (0.06)	1.47–1.80
Weight at diagnosis (kg)		72.41 (13.60)	51.5–114.0	69.64 (14.20)	47.7–127.0	71.12 (13.60)	47.7–127.0
BMI at diagnosis		27.31 (4.81)	17.61–39.02	26.14 (5.02)	17.95–43.84	26.76 (4.92)	17.61–43.84
CDH5 (ng ml^−1^)		9.51 (9.84)	0.12–43.03	13.77 (13.93)	0.03–79.10	11.49 (12.05)	0.03–79.10
HPA (AU ml^−1^)		151.98 (143.56)	3.22–611.27	144.64 (137.34)	11.26–839.78	148.58 (140.11)	3.22–839.78
CDH5/HPA ratio (ng AU^−1^)		0.085 (0.067)	0.003–0.296	0.114 (0.083)	0.001–0.586	0.098 (0.076)	0.001–0.586
		***n***	**%**	***n***	**%**	***n***	**%**
Grade 1		3	5.0	2	3.8	5	4.5
Grade 2	*	22	36.7	20	38.5	42	37.5
Grade 3		35	58.3	30	57.7	65	58.0
Lymph node -ve		26	43.3	22	42.3	48	42.9
Lymph node 1–3 +ve	*	21	35.0	13	25.0	34	30.4
Lymph node >3 +ve		13	21.7	17	32.7	30	26.8
ER positive		44	73.3	32	61.5	76	67.9
ER negative	*	16	26.7	20	38.5	36	32.1
PR positive		20	33.3	18	34.6	38	33.9
PR negative	*	22	36.7	16	30.8	38	33.9
HER2 positive		13	21.7	15	28.8	28	25.0
HER2 negative	*	37	61.7	26	50.0	63	56.3
Vascular Invasion		25	41.7	29	55.8	54	48.2
No vascular invasion		33	55.0	23	44.2	56	50.0
Local recurrence		1	1.7	5	9.6	6	5.4
No local recurrence		59	98.3	47	90.4	106	94.6
Pre-menopausal		14	23.3	15	28.8	29	25.9
Peri-menopausal		15	25.0	11	21.2	26	23.2
Post-menopausal		31	51.7	25	48.1	56	50.0
Blood group A		26	43.3	20	38.5	46	41.1
Blood group B		8	13.3	1	1.9	9	8.0
Blood group AB		6	10.0	4	7.7	10	8.9
Blood group O		20	33.3	27	51.9	47	42.0
Neoadjuvant chemotherapy		3	5.0	9	17.3	12	10.7
No neoadjuvant chemotherapy		57	95.0	43	82.7	100	89.3
Adjuvant Chemotherapy		44	73.3	34	65.4	78	69.6
No adjuvant chemotherapy		16	26.7	18	34.6	34	30.4
Tamoxifen		29	48.3	21	40.4	50	44.6
Arimidex/anastrozole		11	18.3	8	15.4	19	17.0
Herceptin		7	11.7	5	9.6	12	10.7
Other		6	10.1	4	7.6	10	9.0
None		7	11.7	14	26.9	21	18.8
Current smoker		3	5.0	2	3.8	5	4.5
Ex-smoker		20	33.3	14	26.9	34	30.4
Non-smoker		36	60.0	31	59.6	67	59.8
Current drinker		50	83.3	37	71.2	87	77.7
Former drinker		2	3.3	3	5.8	5	4.5
Non-drinker		7	11.7	7	13.5	14	12.5

Abbreviations: BMI=body mass index; CDH5=cadherin-5; ER=oestrogen receptor; HER2=receptor tyrosine-protein kinase erbB-2; HPA=*helix pomatia* agglutinin; NSR=no sign of recurrence; PR=progesterone receptor; REC=recurrent breast cancer. NSR and REC patients were matched according to asterisked categories. Where percentages do not total 100, the absent data was not collected and/or is unknown.
