# Supplementary material for: Cadherin-5: a biomarker for metastatic breast cancer with optimum efficacy in oestrogen receptor-positive breast cancers with vascular invasion
Source: Br J Cancer. 2016 Mar 24;114(9):1019–26. doi: 10.1038/bjc.2016.66 (PMC4984911; doi:10.1038/bjc.2016.66)
Supplement: Supplementary Figure 1 [file bjc201666x1.ppt]

## Slide 1
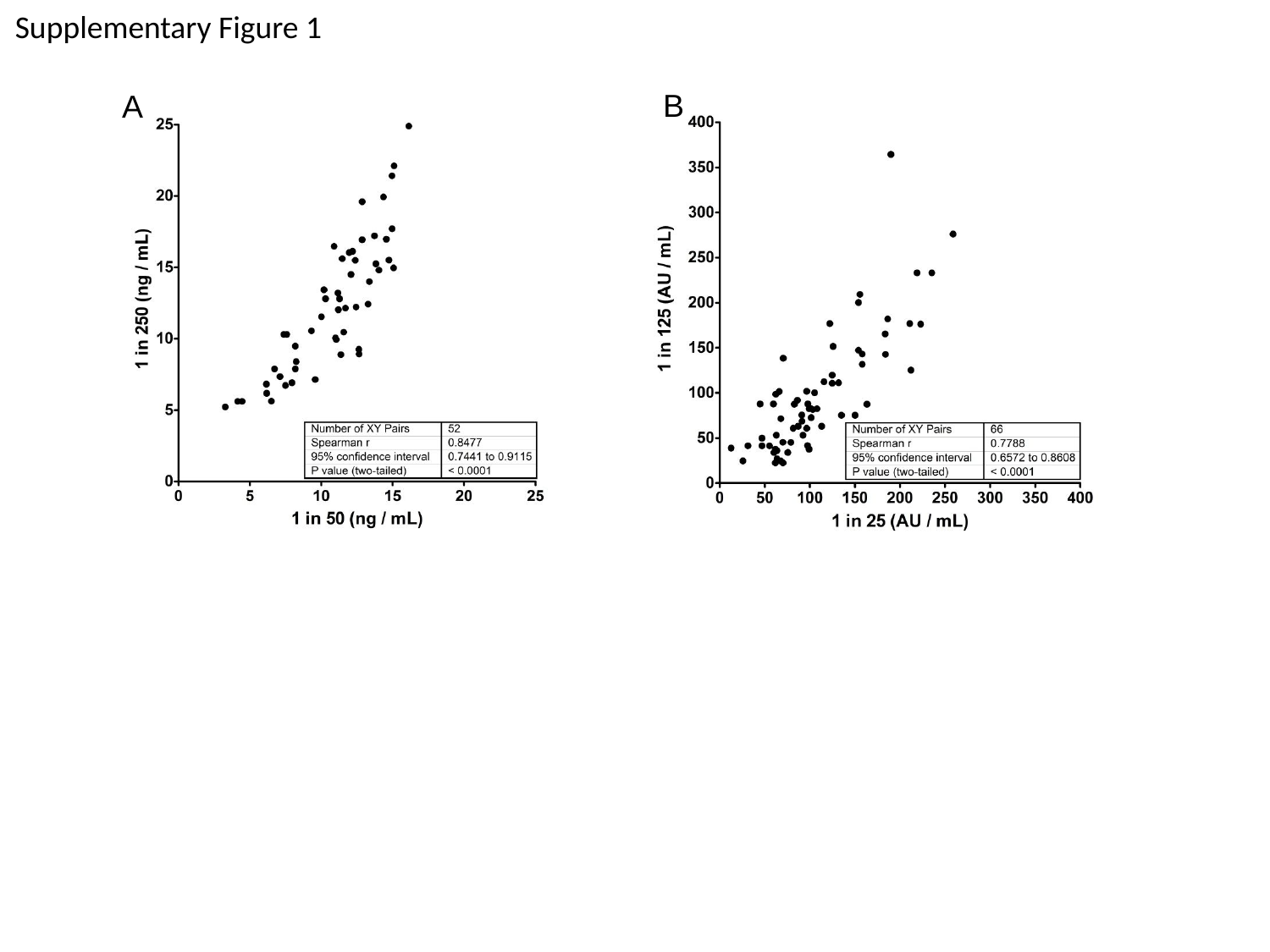

Supplementary Figure 1
B
A

## Slide 2
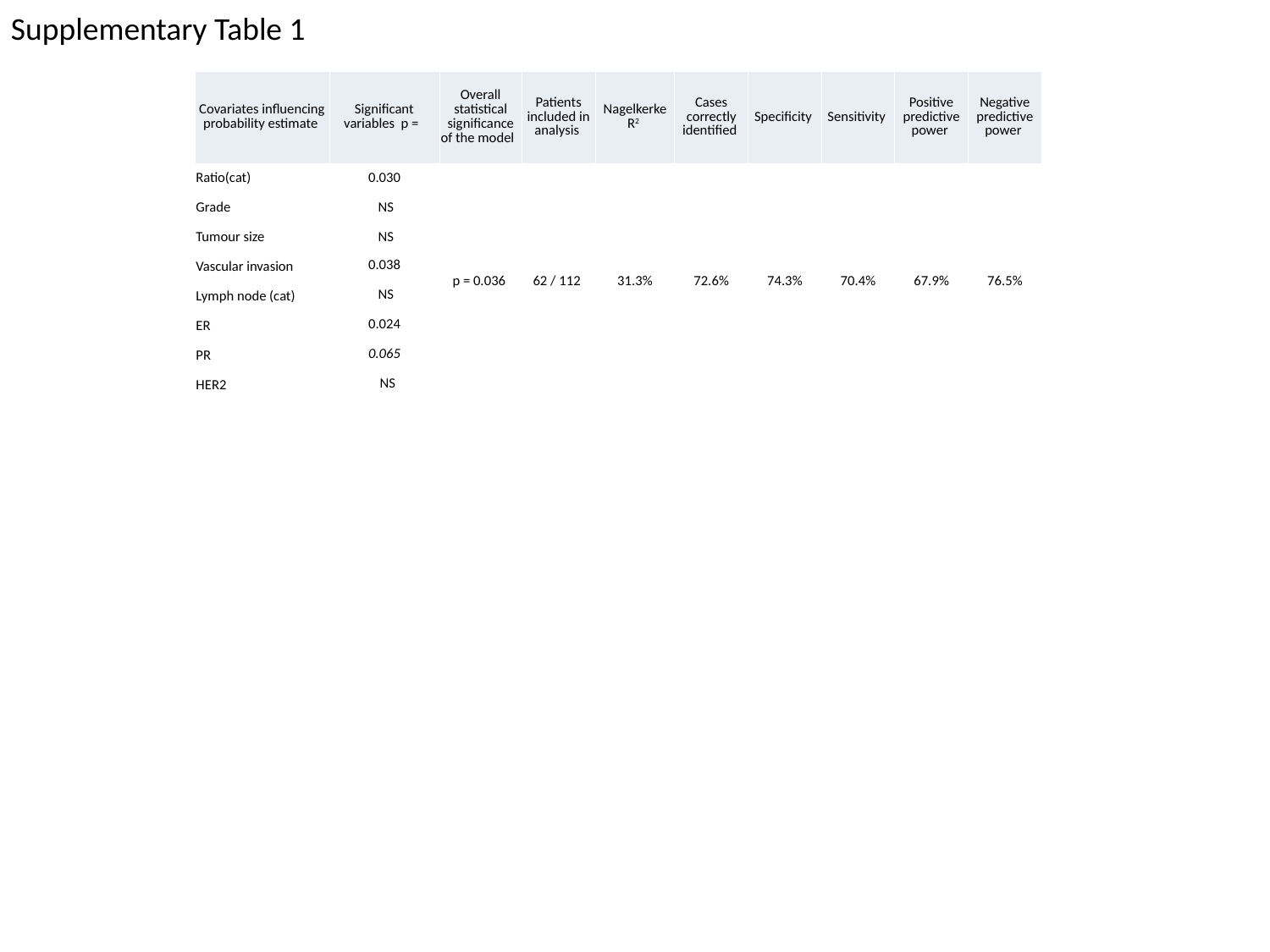

Supplementary Table 1
| Covariates influencing probability estimate | Significant variables p = | Overall statistical significance of the model | Patients included in analysis | Nagelkerke R2 | Cases correctly identified | Specificity | Sensitivity | Positive predictive power | Negative predictive power |
| --- | --- | --- | --- | --- | --- | --- | --- | --- | --- |
| Ratio(cat) | 0.030 | p = 0.036 | 62 / 112 | 31.3% | 72.6% | 74.3% | 70.4% | 67.9% | 76.5% |
| Grade | NS | | | | | | | | |
| Tumour size | NS | | | | | | | | |
| Vascular invasion | 0.038 | | | | | | | | |
| Lymph node (cat) | NS | | | | | | | | |
| ER | 0.024 | | | | | | | | |
| PR | 0.065 | | | | | | | | |
| HER2 | NS | | | | | | | | |
