# Supplementary material for: Cadherin-5: a biomarker for metastatic breast cancer with optimum efficacy in oestrogen receptor-positive breast cancers with vascular invasion
Source: Br J Cancer. 2016 Mar 24;114(9):1019–26. doi: 10.1038/bjc.2016.66 (PMC4984911; doi:10.1038/bjc.2016.66)
Supplement: Supplementary Figure Legends [file bjc201666x2.doc]

**SUPPLEMENTARY FIGURE LEGENDS**

Supp Fig. 1. Correlation between biomarker levels measured at two concentrations as assessed by Spearman's rank correlation coefficient for A) CDH-5 and B) HPA.

Supp Table 1. Binomial logistic regression to identify covariates influencing the probability estimate of developing distant metastasis.
